# Dealing with Challenges Posed by Antimicrobial Resistance in Long-Term Acute-Care Rehabilitation Facilities

**DOI:** 10.3390/antibiotics14020147

**Published:** 2025-02-03

**Authors:** Camilla Grifoni, Marco Coppi, Ilaria Baccani, Alberto Antonelli, Luciana Bevilacqua, Lorenzo Brambilla, Fabio Arena, Roberto Pupillo, Gian Maria Rossolini

**Affiliations:** 1 Severe Brain Injuries Unit, IRCCS Don Gnocchi Foundation, 50143 Florence, Italy; cgrifoni@dongnocchi.it; 2 NARR Joint Laboratory, University of Florence—IRCCS Don Gnocchi Foundation, 50134 Florence, Italy; marco.coppi@unifi.it (M.C.); ilaria.baccani@unifi.it (I.B.); alberto.antonelli@unifi.it (A.A.); 3 Department of Experimental and Clinical Medicine, University of Florence, 50134 Florence, Italy; 4 Microbiology and Virology Unit, Careggi University Hospital, 50134 Florence, Italy; 5 IRCCS Don Gnocchi Foundation, 20148 Milan, Italy; lbevilacqua@dongnocchi.it (L.B.); lbrambilla@dongnocchi.it (L.B.); fabio.arena@unifg.it (F.A.); 6 Department of Clinical and Experimental Medicine, University of Foggia, 71122 Foggia, Italy; 7 Medical Direction, IRCCS Don Gnocchi Foundation, 50143 Florence, Italy; rpupillo@dongnocchi.it

Antimicrobial resistance (AMR) has emerged as one of the major challenges for human health, with a remarkable burden of mortality, morbidity, and healthcare-associated costs. A recent study estimated that around 1.3 million deaths were attributable to antibiotic-resistant bacteria at the global level in 2019, i.e., a dimension comparable to or higher than that attributable to major infectious threats such as malaria, tuberculosis, and HIV/AIDS [1].

Infections caused by antibiotic-resistant pathogens are encountered globally in healthcare settings and in the community. However, due to patient clustering, frequent antibiotic exposure, and other medical practices, healthcare facilities play a major role in the emergence and dissemination of AMR. Indeed, patients admitted to healthcare facilities are at higher risk for infection by antibiotic-resistant pathogens, although with variable rates depending on the epidemiological context and the type of healthcare setting [2].

While acute-care hospitals are well-known hubs for AMR emergence and dissemination, long-term care facilities (LTCFs) have also been increasingly recognized as settings playing a major role in this phenomenon, and patients attending these places are also considered at high risk for infections by antibiotic-resistant pathogens [3]. Indeed, high rates of multidrug-resistant organisms (MDROs), such as methicillin-resistant *Staphylococcus aureus* (MRSA), vancomycin-resistant enterococci (VRE), extended-spectrum β-lactamase-producing *Enterobacterales* (ESBLE), carbapenem-resistant *Enterobacterales* (CRE), carbapenem-resistant *Acinetobacter* (CRAb) and difficult-to-treat *Pseudomonas aeruginosa* (DTR-Pa) have been reported among patients from LTCFs [3,4,5].

However, LTCFs are a heterogeneous group of healthcare structures acting as an interface between hospitals and the community, which may notably differ by the profiles of patients cared for and by the healthcare practices delivered. Consequently, the risk of infection by resistant pathogens may not only reflect the regional AMR epidemiology but also differ according to the LTCF type and even ward within an LTCF [3].

Long-term acute-care rehabilitation hospitals (LTACRHs) are a subset of LTCFs where the risk for infections by MDROs may be particularly high. These facilities, previously classified as type B LTCFs by the European Centre for Disease Prevention and Control, deal with patients with chronic diseases such as multiple sclerosis, dementia, psychiatric diseases, or those who survived severe acute brain injuries (SABIs) that need rehabilitative care within an intensive care context, with an average length of stay (LOS) longer than that encountered in acute-care hospitals [3,6]. In fact, these patients require prolonged neurorehabilitation treatments to regain the highest possible level of physical and cognitive functions. LTACRHs are associated with an outstanding risk for MDRO dissemination and infections for several reasons, including (i) the prolonged time of hospitalization (typically up to one year, which is much longer than the average LOS in intensive care units and medical wards of acute-care hospitals); (ii) the frequent immunocompromised status of patients; (iii) the extensive and prolonged use of invasive medical and rehabilitation devices; (iv) frequent exposure to broad-spectrum antibiotics during hospitalization; (v) challenges with the implementation of structured antimicrobial stewardship programs due to resource constraints and a lack of specific medical expertise; (vi) limited resources and expertise for infection prevention and control (IPC) practices, and challenges with the implementation of some of these practices due to the peculiarities of these healthcare settings (e.g., the need for shared rehabilitation facilities and machineries); and (vii) limited access to diagnostic facilities, which are often outsourced, with the consequent possibility of delayed responses and underdiagnoses [6,7]. Moreover, the frailty and complexity of patients admitted to LTACRHs often require transfer to acute-care hospitals, leading to a ‘revolving door’ phenomenon that contributes to the spread of resistant pathogens among different hospitals [8]. Of the patients requiring admission to LTACRHs, patients with SABIs are among those at the greatest risk of carriage and subsequent development of healthcare-associated infections (HAIs) by MDROs due to their longer LOS, frequent exposure to multiple antimicrobial therapies for prolonged periods, the presence of comorbidities, the need for multiple invasive medical devices including mechanical ventilation, and also immunodeficiency secondary to the acute event, whereby the altered relationship between the Central Nervous System and the Immune System, caused by acute brain events, leads to a syndrome known as CNS injury-induced immunosuppression (CIDS), which increases the risk of infections in this group of patients [9]. In fact, in patients with SABIs, the occurrence of HAI is associated with a significantly lower improvement in physical function, LOS extension, and higher mortality rates [9].

The role that LTACRHs can play in healthcare-related AMR dissemination is underscored by the rates of carriage that MDROs have been reported to have in some settings. For instance, carriage rates of 6% for MRSA [10], 8.6–36.9% for ESBLE [10,11,12], and 1.3–31% for CRE [9,10,13,14,15] have been reported among patients at admission in LTACRHs while rates of 10.2% for ESBLE [16] and 15.7–63% for CRE [9,14,15] have been reported for carriage acquired during hospital stay.

However, data from the literature about AMR prevalence in these contexts are overall limited and not homogeneous since surveillance results are often reported in aggregated form for LTCFs and do not describe the specificity observed in different types of long-term care contexts, including LTACRHs.

Altogether, the above considerations underscore the importance of AMR in the LTACRH context and highlight the existence of unmet needs in this area of healthcare. Some research topics that could be worth addressing are briefly outlined and discussed below.

The first topic of interest concerns the design and evaluation of IPC practices tailored with an LTACRH-specific perspective. IPC practices are essential to contrast the transmission of MDROs within healthcare settings and are typically based on measures to limit the risk of the cross-transmission of MDROs from patients who are infected or colonized by these pathogens (including contact or respiratory precautions, patient isolation, and appropriate hand and environmental hygienic practices) [17,18]. In LTCFs, including LTACRHs, however, it may be difficult to implement some conventional IPC practices, such as contact precautions and patient isolation, due to the need to access common facilities for rehabilitation activities (e.g., gymnasiums, swimming pools). In fact, patient isolation can limit access to rehabilitation activities, hindering rehabilitation programs and negatively affecting clinical outcomes (including a delayed integration into the community). For patients colonized by MDROs, a possible solution could be to deliver selected rehabilitation treatments in the inpatient room, set up a dedicated gymnasium cohort area (box/isolation area), or restrict the access of carriers to dedicated time slots. Moreover, the presence of caregivers and healthcare workers with different cultural backgrounds may be a further challenge to the application of standard IPC measures. In fact, while training in IPC is mandatory for healthcare personnel, it is voluntary for professional caregivers and family members, who can play an essential role in patient care and actively participate in the rehabilitation process, confronting the rehabilitation team with complex patient management decisions [7,19].

Active surveillance to identify carriers of high-risk MDROs (e.g., CRE, DTR-Pa, CRAb, MRSA, VRE, and *Candida auris*) at admission or patients who become colonized by these pathogens during hospitalization is now recommended as part of IPC bundles in several settings, including LTCFs [8,20,21]. However, in LTCFs, including LTACRHs, active surveillance can be delayed by the absence of on-site diagnostic facilities, which are often outsourced and may take a relatively long time to return results; active surveillance results should be obtained rapidly to reduce the time of pre-emptive isolation and maximize the impact of IPC interventions by limiting the risk of cross-transmission events. Altogether, these challenges complicate the enforcement and may reduce the impact of active surveillance practices in rehabilitation settings, highlighting the urgent need for the development and testing of innovative strategies better tailored to these settings. From this perspective, the adoption of rapid and highly automated molecular screening methods for resistant pathogens, which provide results within a few hours and are amenable to use as near-patient solutions, could provide an advantage in these settings by reducing the time of pre-emptive isolation, which could override the higher cost of testing [22]. A similar strategy might be more beneficial in settings with a higher endemicity of resistant pathogens, especially in LTACRHs, where the risk for AMR spread is particularly high. Systematic active surveillance, based on screening for carriage at admission and at variable intervals (if possible on a biweekly basis), should be prioritized for MDROs which are prevalent in each setting and which are known to be associated with higher risks for infection following carriage (e.g., CRE), while point prevalence surveys aimed at monitoring the spread of less common MDROs could be planned and executed at longer intervals to monitor these pathogens as well [7,8]. Indeed, efficient and accurate diagnostic support is crucial for contrasting AMR in LTACRHs, not only for the active surveillance of MDROs but also for the timely diagnosis of MDRO infections, which is essential for antimicrobial stewardship (AS) to reduce the frequency and duration of empiric antimicrobial chemotherapy, and for the enforcement of adequate IPC practices. The availability of data from diagnostic microbiology is also essential for the retrospective surveillance of infections and carriage by MDROs, which should be provided by the referral diagnostic laboratory as an essential component of IPC practices for monitoring trends and the efficacy of interventions [17]. When LTACRHs are missing on-site diagnostic laboratories and outsource diagnostic activities to large private providers, this may lead to delays in the diagnosis of infections, depending on the variable organization of the laboratories (e.g., opening and sample processing times) and the timeline of sample transport systems.

Designing and testing novel diagnostic solutions based on rapid point of care technologies (POCTs) for the diagnosis of certain infections or for the screening of MDRO carriage, supported by external microbiological consulting service for the interpretation of results and proper handling of quality controls appears to be another research topic of interest in this healthcare area. The access to diagnostic microbiology could be adapted according to the infection risk level. For critical care patients, such as those with sABI, microbiological diagnosis should be supported by a 24 h/7 d or 12 h/7 d service for invasive infections and, if possible, the use of rapid diagnostic technologies. In fact, in the case of worsening patient conditions, the likelihood of being transferred to an acute care hospital increases, with the potential impairment of the rehabilitation plan.

A third research topic of interest in this area of healthcare concerns the design and testing of antimicrobial stewardship programs (ASPs) tailored for LTACRHs. ASPs include the implementation of dedicated teams, which are already a standard of care in acute-care hospital settings [23] and are also increasingly required in the post-acute rehabilitation phase, now considered a continuum of the acute phase. However, the implementation of such interventions is still limited in the LTCF setting. In fact, approximately 30% of LTCFs participating in the 2016-17 PPS reported that none of the core antimicrobial stewardship elements included in the survey were adopted [3]. In fact, ASPs need multidisciplinary teams, including experts in infectious diseases, clinical microbiology, clinical epidemiology, and clinical pharmacology, which may be more challenging to organize in LTCF settings due to the lack of specific consultants. Consequently, in similar settings, ASP must be adapted to the needs of the specific department and to the local epidemiology while addressing challenges related to the limited access to novel and high-cost antimicrobials and the lack of dedicated infectious disease and other consultants [24]. Indeed, a previous study on the implementation of a targeted ASP in a large neurorehabilitation setting was successful in promoting responsible antibiotic use and in reducing AMR rates and the incidence of *Clostridioides difficile* infections [5], underscoring the importance of efforts in this area. Key elements of the program included increased compliance with prescribing appropriate antibiotics, optimizing the treatment of non-severe urinary infections, and limiting the use of carbapenems in empiric therapy. Small-group meetings were held between physicians and nurses, discussing the epidemiology of antimicrobial resistance, the appropriateness of antibiotic prescribing, and infection control measures.

In conclusion, LTACRHs are healthcare settings at high risk for MDROs, especially in areas of high prevalence of AMR, and research in the implementation of dedicated IPC bundles, ASP, and diagnostic stewardship programs is essential to ensure safer and higher quality care to reduce the selective pressure of antibiotics, improve the efficiency of rehabilitation pathways, and optimize resources.
